# A Double Blind, Placebo-Controlled, Randomized Crossover Study of the Acute Metabolic Effects of Olanzapine in Healthy Volunteers

**DOI:** 10.1371/journal.pone.0022662

**Published:** 2011-08-09

**Authors:** Vance L. Albaugh, Ravi Singareddy, David Mauger, Christopher J. Lynch

**Affiliations:** 1 Department of Cellular and Molecular Physiology, The Pennsylvania State University College of Medicine, Hershey, Pennsylvania, United States of America; 2 Department of Psychiatry, The Pennsylvania State University College of Medicine, Hershey, Pennsylvania, United States of America; 3 Department of Public Health Sciences, The Pennsylvania State University College of Medicine, Hershey, Pennsylvania, United States of America; University of Tor Vergata, Italy

## Abstract

**Background and Rationale:**

Atypical antipsychotics exhibit metabolic side effects including diabetes mellitus and obesity. The adverse events are preceded by acute worsening of oral glucose tolerance (oGTT) along with reduced plasma free fatty acids (FFA) and leptin in animal models. It is unclear whether the same acute effects occur in humans.

**Methodology/Principal Findings:**

A double blind, randomized, placebo-controlled crossover trial was conducted to examine the potential metabolic effects of olanzapine in healthy volunteers. Participants included male (8) and female (7) subjects [18–30 years old, BMI 18.5–25]. Subjects received placebo or olanzapine (10 mg/day) for three days prior to oGTT testing. Primary endpoints included measurement of plasma leptin, oral glucose tolerance, and plasma free fatty acids (FFA). Secondary metabolic endpoints included: triglycerides, total cholesterol, high- and low-density lipoprotein cholesterol, heart rate, blood pressure, body weight and BMI. Olanzapine increased glucose Area Under the Curve (AUC) by 42% (2808±474 *vs.* 3984±444 mg/dl·min; P = 0.0105) during an oGTT. Fasting plasma leptin and triglycerides were elevated 24% (Leptin: 6.8±1.3 *vs.* 8.4±1.7 ng/ml; P = 0.0203) and 22% (Triglycerides: 88.9±10.1 *vs.* 108.2±11.6 mg/dl; P = 0.0170), whereas FFA and HDL declined by 32% (FFA: 0.38±0.06 *vs.* 0.26±0.04 mM; P = 0.0166) and 11% (54.2±4.7 vs. 48.9±4.3 mg/dl; P = 0.0184), respectively after olanzapine. Other measures were unchanged.

**Conclusions/Significance:**

Olanzapine exerts some but not all of the early endocrine/metabolic changes observed in rodent models of the metabolic side effects, and this suggest that antipsychotic effects are not limited to perturbations in glucose metabolism alone. Future prospective clinical studies should focus on identifying which reliable metabolic alterations might be useful as potential screening tools in assessing patient susceptibility to weight gain and diabetes caused by atypical antipsychotics.

**Trial Registration:**

ClinicalTrials.gov NCT00741026

## Introduction

The advent of atypical antipsychotics represented a significant improvement in the treatment of schizophrenia and other psychoses, with these newer drugs lacking most of the debilitating movement side effects of first generation compounds. Over the last decade it has become clear that these second generation drugs possessed other significant adverse effects with chronic treatment. The adverse effects are in the endocrine-metabolic and body weight categories. They include body weight gain, increased adiposity, insulin resistance, diabetes, and dyslipidemia [1], [2], [3], [4], [5], [6], [7], [8]. The close association of these metabolic sequelae with atypical antipsychotics, a popular and multi-billion dollar per year drug class, has led to efforts to further characterize the adverse events in order to discover new ways to screen or prevent the resulting obesity and diabetes, which have already reached epidemic proportions in the general population [9], [10], [11].

Understanding the mechanism of these side effects has been challenging. Most clinical studies seeking to elucidate these mechanisms have been conducted in patient samples after months to years of chronic treatment with antipsychotics. Mechanistic insights into the cause of the obesity and diabetes in such studies have to take into account the multiple potential sources of the effects including the direct actions of the drugs as well as the differential drug effects on weight gain, food intake, physical activity and other co-morbid conditions frequently associated with mental illness, e.g. [12], which may have genetic and/or lifestyle factors [13], [14], [15]. In longer-term studies, it is more difficult to ascertain which effects are secondary to obesity and diabetes and which are primarily caused by the atypical antipsychotics. For example one finding from chronic studies has been that olanzapine decreases FFA despite its ability to also cause diabetes or insulin resistance [16], [17]. This is paradoxical because diabetes and insulin resistance are normally associated with elevated FFA not lower FFA. Despite these challenges, a considerable amount of basic and clinical research has focused on unraveling the molecular mechanisms of these adverse effects. Since the most severe diabetes and obesity in patients are observed with olanzapine and clozapine therapy, reviewed in refs: [18], [19], these two compounds have, understandably, become the focus of much of the work in animal models.

Some of the chronic metabolic effects of these drugs, such as increased plasma glucose and decreased FFA, occur rapidly in animal models and thus imply that they could precede as opposed to follow the development of obesity. For example, several atypical antipsychotics, including olanzapine, have been shown to exhibit rather early and sustained metabolic and endocrine effects beginning within minutes to hours following drug administration [20], [21], [22], [23]. This time course could be important because if these effects occur early before confounding factors set in, it would improve the chances of understanding the underlying mechanism. A caveat is that recent data from animal studies have raised a number of questions about the relevance of animal models to the clinical problem. For example, in contrast to humans, drug-induced hyperphagia in response to olanzapine is sexually dimorphic in rat models, with female rats exhibiting robust increases in food intake and body weight gain in contrast to males which do not show this robust effect [24], [25], [26], [27]. In fact, clozapine fails to cause hyperphagia in either female or male rodents. Nevertheless, both drugs increase visceral adiposity, impair glucose tolerance, and decrease lean muscle mass in rodent models [20], [23], [28].

Clinical studies validating these acute effects are lacking, especially because of the challenge of isolating these effects in a schizophrenic population. However, such information would help us understand if animal models accurately reflect human physiology in this regard. If so, this would provide a reasonable rationale to support subsequent studies to determine the mechanisms of these effects and their relevance to the development of obesity and diabetes. This would effectively allow researchers to better define the acute metabolic effects in human subjects in hopes of validating animal studies, while at the same time focusing the direction of future laboratory efforts on biology that translates to the bedside. Therefore, we decided to examine whether some of the acute metabolic changes that have been observed in rodents treated with atypical antipsychotics also occurred in humans.

In the following report we demonstrate that acute (3 day) olanzapine administration in healthy volunteers is associated with changes in metabolic parameters, including impaired glucose tolerance, increased triglycerides, and decreased high-density lipoprotein cholesterol without changes in low-density lipoprotein or total cholesterol levels. Evidence suggests that drug-induced changes in plasma leptin concentration, whose potential decrease had been hypothesized to trigger hyperphagia in rats, may not be clinically relevant. Finally, olanzapine was associated with decreased circulating free fatty acids, a finding described in recent clinical and animal studies, which may give fresh insight to the mechanism of these metabolic side effects.

## Materials and Methods

The protocol for this trial and supporting CONSORT checklist are available as supporting information; see Checklist S1 and Protocol S1.

### Ethics Statement

The institutional review board of Penn State University College of Medicine approved the study, including the protocol, scientific rationale, subject safety, recruitment fliers and other study advertisements. The study, *A Double-blind, Placebo-controlled, Crossover Study Examining the Acute Effects of Olanzapine on Plasma Leptin, Glucose Tolerance and Free Fatty Acids in Healthy Volunteers*, was registered online as part of the National Institutes of Health clinical trials database (Clinicaltrials.gov registration number NCT00741026).

### Participants

All healthy subjects were recruited by IRB-approved fliers and screened for eligibility prior to enrollment in the study. Volunteers 18 to 30 years of age with body mass indices of 18.5 to 25 kilograms per square meter with the ability to swallow tablets and give informed consent were eligible for the study. Subjects who met any of the following criteria were excluded: (1) Any DSM-IV TR Axis I psychiatric disorder (except nicotine dependence); (2) Presence of a medical disorder that may confound the assessment of relevant biologic measures, including: significant organ system dysfunction, metabolic diseases, type 1 diabetes mellitus, type 2 diabetes mellitus, pregnancy, endocrine disease, coagulopathy, clinically significant anemia, or acute infection; (3) Subjects who have taken any antipsychotic medication within the last 6 months; (4) Personal or family history of seizures or cardiac arrhythmias. A total of 42 individuals were screened between July 2008 and June 2009, and of those individuals 15 subjects were eligible based on inclusion/exclusion criteria.

### Study Design and Interventions

The study was conducted at the General Clinical Research Center (GCRC) of the Milton S. Hershey Medical Center / Penn State Hershey College of Medicine. There were no major changes to the study protocol after initiation of the study. Given the considerable variation in human research subjects, we opted for a crossover design in which each subject would serve as his/her own control to improve sensitivity for detection of acute hormonal and/or metabolic changes. Treatment sequence assignment was determined using blocked randomization (block size = 4) to ensure balance across the study recruitment period. The SAS statistical software system version 9.1 (SAS Inc. Cary, NC) was used to program the randomization algorithm. The Investigational Drug Service at the Milton S. Hershey Medical Center prepared blinded olanzapine (Eli Lilly, USA) and placebo tablets according to the randomization algorithm. The subjects participating in the study, the study investigators, and the nursing staff in the GCRC were blind to the intervention type until the conclusion of the study. The only exception to this was one episode requiring un-blinding of a subject who developed orthostatic hypotension 24 h after completing the study.

Written informed consent was obtained from all qualifying subjects prior to the beginning of the study. The staff in the GCRC at Penn State handled all subject recruitment. Subjects were randomized to receive three days of oral olanzapine (10 mg/day) or placebo in a double-blind fashion. This dose of olanzapine was used because it is within the range of commonly used starting doses in patients receiving olanzapine for psychotic symptoms and is comparable to previous studies in healthy volunteers [16], [29], [30]. All subjects self-administered one study tablet per evening for three consecutive evenings. On the morning following the final dose of olanzapine or placebo, the subjects reported to the GCRC after an overnight fast for blood collection and an oral glucose tolerance test. Volunteers had a minimum of two weeks for ‘wash-out’ between crossovers and returned within four weeks of completing the first arm of the trial for their second visit to complete the study. Thus, subjects completed both arms of the trial within approximately four weeks.

The primary study endpoint of the study was leptin concentration, placebo compared to olanzapine treatment, which was based on previous rodent studies [25], [31]. Secondary metabolic endpoints included: oral glucose tolerance, free fatty acids, triglycerides, total cholesterol, and high- and low-density lipoprotein cholesterol, heart rate, blood pressure, body weight and BMI.

### Study Follow-Up and Adverse Events

Participants in the trial were followed-up via telephone call approximately 4–6 weeks after completion of the study and screened for additional side effects or changes in their health that they had experienced since their participation. There were no unanticipated adverse events during the study; however, there were several anticipated side effects observed in the olanzapine arm likely related to active drug treatment. These anticipated effects included the one episode of orthostatic hypotension that required un-blinding, as well as six episodes of volunteers subjectively reporting feeling “tired”, “fatigued” or “worn-down”. Individuals also reported having “dry mouth” during the study arm that corresponded to active drug treatment.

### Oral Glucose Tolerance Test

Subjects were given explicit instructions and patient handout information on how to consume a high carbohydrate diet throughout the 3-day duration for each of the study arms. Subjects refrained from exercising (i.e. aerobic or anaerobic) during each of the three-day periods prior to the glucose tolerance testing. All oral glucose tolerance testing was conducted in the morning. These tests were conducted at the same time of the morning to minimize variability because of known circadian influences on glucose homeostasis and insulin secretion [32], [33]. After an overnight fast of approximately 10–12 h, baseline blood samples were collected. Subsequently, subjects drank a bolus solution containing 75 g of glucose (Fisher Health care: FisherBrand 75 SUN-DEX, cat # 401223FB), at which the time was recorded as zero (t = 0). Serial blood samples were then collected every 30 minutes for two hours (t = 30, 60, 90, 120). Blood samples were immediately centrifuged at 1,800*× g* for 10 minutes and plasma and serum frozen for future analysis.

### Metabolite and Hormone Assays

Plasma glucose concentrations were measured using glucose oxidase membranes (Yellow Springs Instrument Company, Yellow Springs, Ohio). Plasma insulin and leptin concentrations were measured using commercially available double-antibody radioimmunoassays (Millipore, Billerica, MA). Blood lipid panels (i.e. total cholesterol, low-density lipoprotein cholesterol, high-density lipoprotein cholesterol, triglycerides) and free fatty acid concentrations were determined enzymatically and spectrophotometrically (Quest Diagnostic Labs; Camp Hill, Pennsylvania). To limit inter-assay variability, samples from each subject's placebo and active treatment arms were assayed together after completion of the study. Leptin assays were completed in total at the conclusion of the study.

### Statistical Methods

For all results, data are summarized numerically with mean and standard deviation and displayed with standard box plots. The study was primarily powered (90% Power) to detect a difference in serum leptin concentration between the placebo and olanzapine arms of the crossover study. Power and sample size calculations were based on previous studies [25], [31] using the method of Senn [34], and calculated using a conservative estimate of within-subject correlation and assuming a modest correlation benefit for the crossover design. Wilcoxon signed-rank tests were used to calculate significant differences (P<0.05) compared to placebo treatment. All statistical analyses were completed with GraphPad Prism and/or InStat computer software (GraphPad Software, San Diego, CA).

## Results

### Subject Characteristics and Anthropometric Data

**Figure 1:**
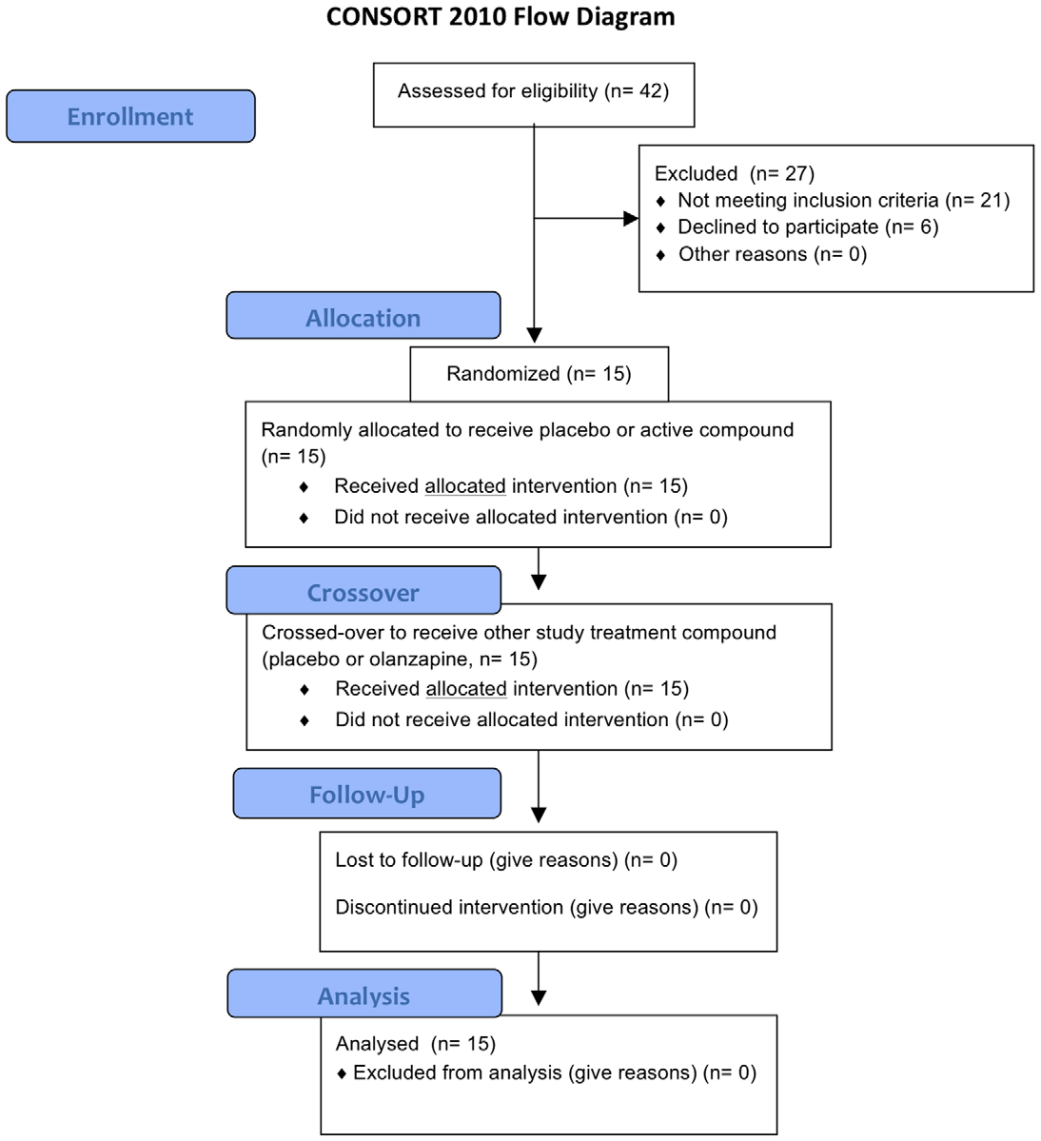
CONSORT 2010 Flow Diagram. The schema graphically outlines the design and conduct of the clinical study.

We conducted a double blind, randomized, placebo-controlled trial to detect acute metabolic effects of olanzapine in healthy volunteers. The CONSORT 2010 flow diagram of this study is shown in Fig. 1. Of the individuals screened, fifteen individuals (8 males and 7 females) began the trial, though one individual dropped out of the study during the final visit to complete the study. This was due to emesis which the volunteer experienced after beginning the oral glucose tolerance test, which took place after all other laboratory samples for analysis had been collected. Thus, data from this participant is included for all study endpoints except glucose tolerance testing. The remaining fourteen individuals that began the trial completed both study arms in a double blind, crossover, and randomized fashion according to the approved study protocol.

The study was designed to enroll a total of sixteen healthy volunteers and for each volunteer to complete both study arms (i.e. placebo and olanzapine) prior to stopping the study, un-blinding, and beginning data analysis. However, the study was stopped prematurely secondary to lack of funding after only recruiting fifteen eligible study subjects. Nevertheless, after officially stopping the study it was decided to un-blind the data collected to date from the participants that had completed the study for analysis.

The average age of the subjects was 26.6±0.5 years. When analyzing sexes separately, the mean ages of male and female subjects did not differ significantly (27.0±0.6 *vs.* 26.2±1.0 years, respectively). Neither bodyweight nor BMI differed significantly in either treatment arm when placebo and olanzapine arms of the study were compared overall or with respect to sex (Table 1). Other baseline characteristics that were measured included heart rate and systolic and diastolic blood pressures, none of which differed between treatment arms when analyzed together or with respect to sex.

**Table 1:** Subject characteristics following placebo and olanzapine treatment.

Sex	Condition	Weight (kg)	BMI (kg/m^2^)	Heart Rate (bpm)	Systolic BP (mmHg)	Diastolic BP (mmHg)
Total	Placebo	68.2±2.9	23.0±0.5	69.9±2.6	113.3±2.8	68.3±2.0
(n = 15)	Olanzapine	68.9±3.0	23.0±0.6	67.6±3.5	111.7±3.1	69.5±1.8
Male	Placebo	76.5±2.6	23.1±0.7	67.9±4.2	118.5±4.0	72.0±0.8
(n = 8)	Olanzapine	77.7±2.6	23.3±0.8	63.0±4.5	115.3±5.1	71.4±2.7
Female	Placebo	58.7±2.0	22.8±0.8	72.3±3.1	107.3±2.7	64.0±3.6
(n = 7)	Olanzapine	58.8±2.0	22.6±0.8	72.9±5.2	107.6±2.9	67.4±2.3

Results are expressed as the mean ± S.E.

### Effects of Olanzapine on Leptin and Glucose Tolerance

**Figure 2:**
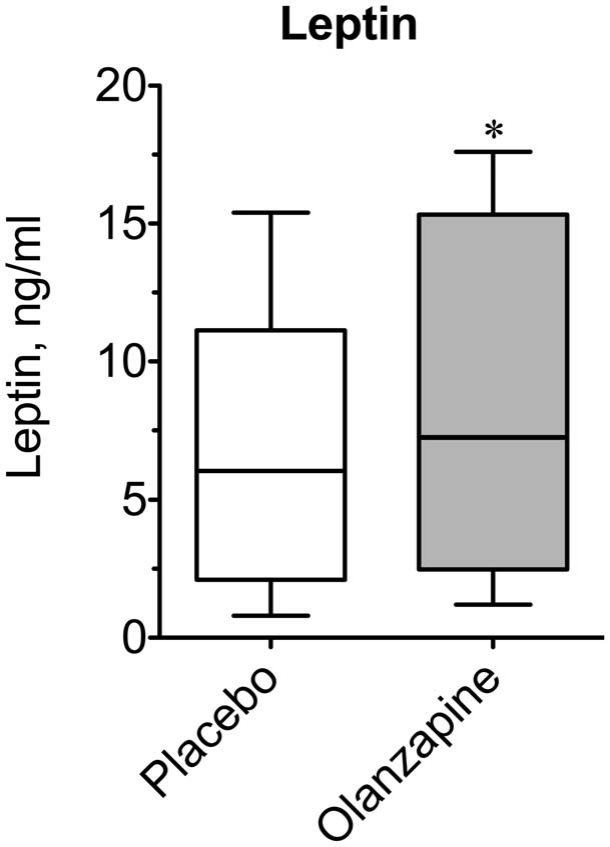
Effect of olanzapine on plasma leptin. Baseline fasting blood samples were drawn approximately 10–12 h following the final dose of olanzapine or placebo tablets. Plasma leptin was measured at the conclusion of the study for each subject under placebo- and olanzapine-treated conditions. Data are expressed as a box plot for placebo and olanzapine-treated subjects. An asterisk indicates (P = 0.02) a significant difference between the medians of the placebo and olanzapine groups using a Wilcoxon matched-pairs signed rank test (n = 15).

Leptin concentrations increase with antipsychotic-induced weight gain as expected with increased adiposity [35], [36], though we demonstrated that olanzapine decreased circulating leptin in rats prior to the onset of hyperphagia and adiposity, suggesting that it may have triggered this response [20], [25]. It is not clear whether this occurs clinically, but it is feasible that a disproportionately low leptin for a given degree of adiposity could drive increased food intake by patients [37]. However, in the current study median fasting leptin (Fig. 2) was not decreased, but rather increased by 24% following olanzapine treatment (6.8±1.3 *vs.* 8.4±1.7 ng/ml; P = 0.0203), suggesting that attenuated leptin is not involved in drug-induced hyperphagia.

**Figure 3:**
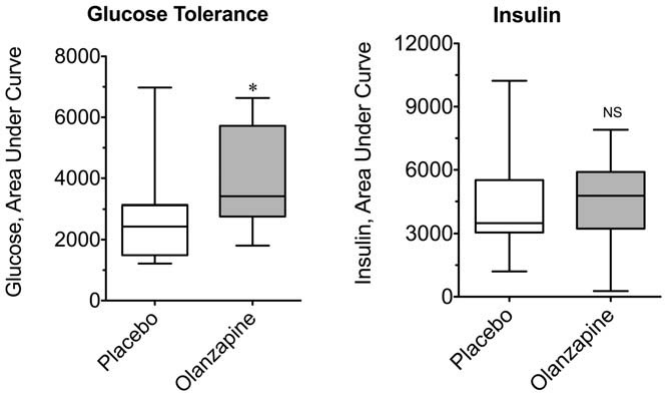
Effects of olanzapine on the glucose and insulin responses during oral glucose challenge. Blinded olanzapine or placebo tablets were self-administered by healthy volunteers for three days prior to conduction of a standard oral glucose tolerance test. In the morning, approximately 10–12 h following the final dose of placebo or drug compound, baseline blood samples were collected and then volunteers self-administered an oral glucose-containing solution. Serial blood samples were drawn at 30 min intervals for two hours. Plasma glucose and insulin concentrations were determined for each time point of the tolerance test. Area under the curve for (A) Glucose and (B) Insulin were calculated for each individual oral glucose tolerance test under placebo and active drug conditions. Data are expressed as box plots for the placebo and olanzapine-treated subjects. An asterisk indicates (P = 0.011) a significant difference between the medians of the placebo and olanzapine groups using a Wilcoxon matched-pairs signed rank test (n = 14).

Atypical antipsychotics are diabetogenic drugs [2], [3], [7], though the rapidity of changes to glucose tolerance and/or insulin sensitivity in patients is unknown. Animal models [20], [22], [25], [38], [39] have demonstrated that changes in glucose tolerance and insulin sensitivity are detectable within hours to days following drug administration, prior to the onset of hyperphagia or detectable weight gain. Oral glucose tolerance testing following three days of olanzapine or placebo treatment in healthy volunteers (Fig. 3A) revealed that the median area under the curve (AUC) for glucose was increased by 42% following olanzapine treatment (2808±474 *vs.* 3984±444 mg/dl·min; P = 0.0105). The AUC insulin (Fig. 3B) did not differ between the placebo and olanzapine treatment (4390±664 *vs.* 4507±579 µU/ml·min), though there was a trend for higher plasma insulin values in the olanzapine group (12.7±1.0 vs. 14.5±1.1 µU/ml, n = 15).

### Effects of Olanzapine on Blood Lipids and Cholesterol

**Figure 4:**
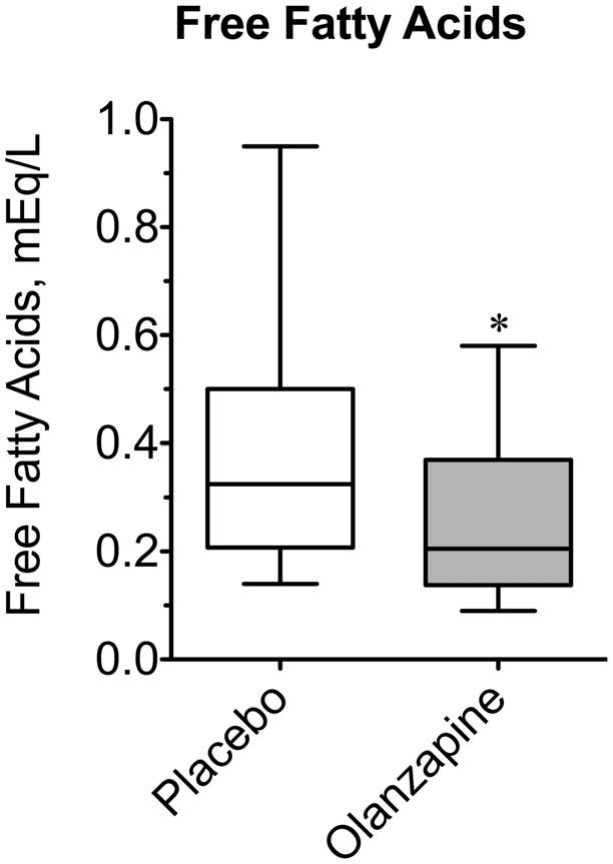
Effect of olanzapine on plasma fasting free fatty acids. Baseline blood samples were collected approximately 10–12 h following the final dose of olanzapine or placebo tablets prior to beginning an oral glucose tolerance test for hormone and metabolite analyses. Plasma free fatty acid concentrations were measured for all subjects under placebo- and olanzapine-treated conditions. Data are expressed as a box plot for placebo and olanzapine-treated groups. An asterisk indicates (P = 0.016) a significant difference between the medians of the placebo and olanzapine groups (n = 15).

In addition to insulin resistance and body weight gain, olanzapine and other antipsychotics are associated with dyslipidemia, a known risk factor for coronary artery disease [40]. To characterize the potential effects of olanzapine on other metabolites associated with insulin resistance and the metabolic syndrome, circulating concentrations of free fatty acids, triglycerides, and cholesterol (i.e. Total, HDL, LDL) were measured following each treatment arm. Insulin resistant states, like obesity and diabetes, are typically associated with higher rather than lower concentrations of FFAs, secondary to decreased insulin-mediated suppression of lipolysis from adipose stores. As mentioned earlier chronic olanzapine has been paradoxically reported to decrease FFA. Consistent with the findings in chronic studies, median free fatty acid concentration (Fig. 4) was ∼36% lower following acute olanzapine treatment (0.38±0.06 *vs.* 0.26±0.04 mM; P = 0.0166).

**Figure 5:**
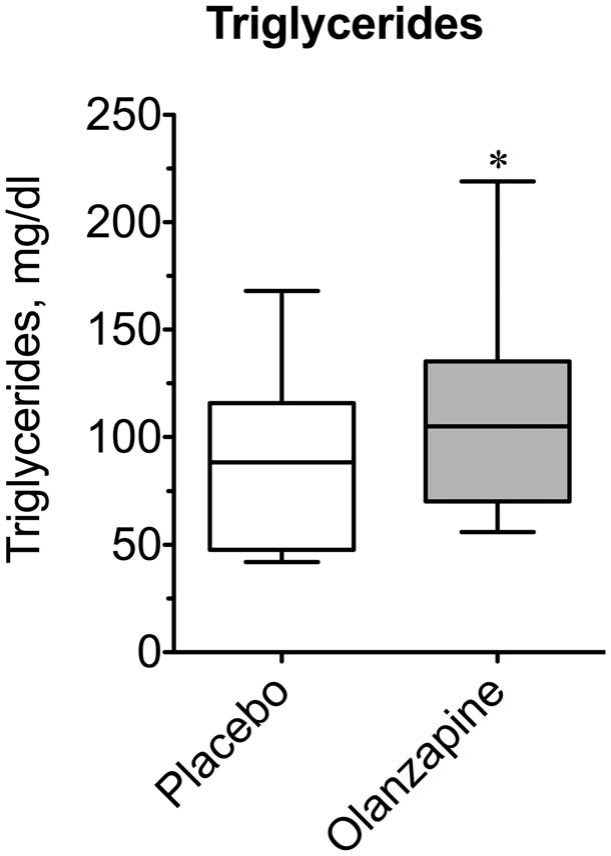
Effect of olanzapine on plasma triglycerides. For each subject baseline blood samples were drawn 10–12 h following the final dose of placebo or olanzapine tablets prior to beginning an oral glucose tolerance test for hormone and metabolite analyses. Plasma triglyceride concentration was measured for all subjects under placebo- and olanzapine-treated conditions. Data are expressed as a box plot for placebo and olanzapine-treated groups. An asterisk indicates (P = 0.017) a significant difference between the medians of the placebo and olanzapine groups (n = 15).

**Figure 6:**
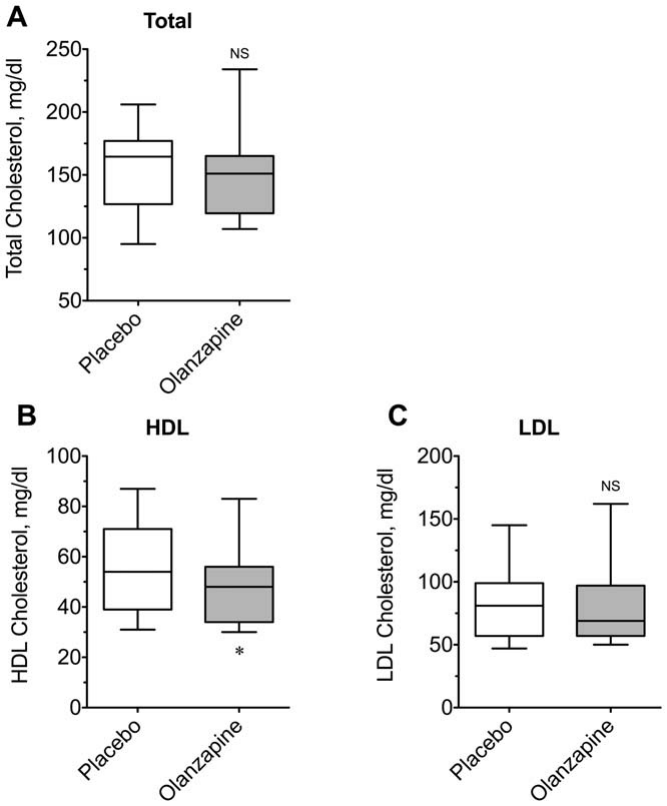
Effect of olanzapine on plasma cholesterol. Plasma concentrations of (A) Total cholesterol, (B) High-density lipoprotein, and (C) Low-density lipoprotein cholesterol were measured for all subjects under placebo- and olanzapine-treated conditions. Data are expressed as box plots for comparison of placebo and olanzapine-treated groups. An asterisk indicates (P = 0.018) a significant difference between the medians of the placebo and olanzapine groups. NS = not significantly different (n = 15).

Given the prevalence of cardiovascular risk factors known to occur in schizophrenic patients [41], [42], we measured cholesterol and triglyceride concentrations in our healthy subjects to determine any baseline effects of olanzapine (Figs. 5 and 6). Neither total cholesterol (156.8±9.1 *vs.* 152.6±10.1 mg/dl), nor did the low-density lipoprotein fraction differ between placebo and olanzapine treatment (82.7±6.6 *vs.* 81.7±7.8 mg/dl). However, median high-density lipoprotein cholesterol was modestly decreased by 10% in the olanzapine group (54.2±4.7 vs. 48.9±4.3 mg/dl; P = 0.0184). Furthermore, the median triglyceride concentration was increased by 22% (88.9±10.1 *vs.* 108.2±11.6 mg/dl; P = 0.0170) after olanzapine treatment. Together these changes translated into a ∼14% increase in the TG∶HDL ratio, a sensitive marker of insulin resistance (P = 0.010). Thus, acute olanzapine administration appears to be associated with negative changes in blood lipid profiles even in otherwise healthy subjects.

## Discussion

In this study we have shown that olanzapine treatment is associated with metabolic effects that are detectable as early as the third treatment day, effects that may be involved in the obesigenic and diabetogenic propensities of olanzapine and other atypical antipsychotics. These metabolic effects included an elevated AUC glucose during oral glucose tolerance testing, consistent with alterations in whole-body insulin sensitivity and/or pancreatic beta-cell secretion of insulin. Secondly, olanzapine was associated with increased concentrations of leptin and triglycerides, factors consistently elevated in obesity and diabetes. Paradoxically, free fatty acids concentrations, which are typically elevated in insulin resistant states, were actually found to be lower following olanzapine treatment. Finally, there was a small but significant decrease in high-density lipoprotein cholesterol in the olanzapine study arm, in the absence of changes in total and low-density lipoprotein cholesterol. To our knowledge, this is the first study to demonstrate such rapid metabolic effects of any of the atypical antipsychotics in humans. Importantly, the results of this study are comparable to and extend previous observations in clinical and animal studies.

### Early Effects on Leptin, Glucose Tolerance, and FFA

The initial aims of the study were to determine if olanzapine had early effects on leptin, oral glucose tolerance, and FFA in otherwise healthy volunteers. Clinical studies have demonstrated that leptin increases with chronic olanzapine treatment [35], [43], [44], though this is expected as circulating leptin increases with increased adipose tissue mass. In several animal models, though, we have observed ∼50% reductions in plasma leptin after acute olanzapine administration [20], [25]. Thus, it is reasonable to speculate that decreases in circulating leptin, a powerful regulator of food intake and body weight, may underlie the eventual increased adiposity and weight gain seen clinically. In our human subjects, however, we detected a small but statistically significant increase in leptin (∼20%). This suggests that our efforts to investigate the mechanism of leptin lowering in the rodents, while of interest from a basic science perspective, might not be relevant to humans, as increased serum leptin likely does not underlie the increased hunger observed in patients taking olanzapine.

In addition to examining potential alterations in leptin, the effects of atypical antipsychotics on insulin sensitivity in healthy volunteers have been examined in a small number of studies using hyperinsulinemic-euglycemic clamp methodology, the ‘gold-standard’ for measuring whole-body insulin sensitivity. As mentioned, clamp studies in animals have demonstrated rapid induction of insulin resistance by olanzapine and other atypical antipsychotics [20], [21], [22], though studies following longer periods of drug administration in healthy volunteers have yielded conflicting results. Sowell and colleagues [29] failed to detect altered insulin sensitivity following three weeks of olanzapine treatment. More recently, though, others have shown that olanzapine decreases insulin sensitivity in healthy males after either eight [16] or ten days of administration [45]. The current study detected an increase in the AUC glucose, consistent with decreased whole-body insulin sensitivity, using the same dose of olanzapine (10 mg/day) as each of these previous human clamp studies. Of note, though, studies in animals have actually shown that olanzapine and other antipsychotics decrease insulin sensitivity within the first hour and possibly within minutes following drug administration. Given the rapid onset of these effects, it would make sense to conduct further studies along the same time course in healthy human subjects to further validate the animal models.

Recent studies in human subjects have demonstrated that chronic olanzapine is associated with decreased circulating free fatty acid concentrations [16], [17]. Interestingly, lower FFA concentrations were consistently observed in the olanzapine arm of the current study, despite higher concentrations of FFA being more closely associated with impaired glucose tolerance and insulin resistance. The mechanism of this FFA lowering effect is currently unknown, though in recent studies from our lab, olanzapine increased fatty acid oxidation in peripheral tissues and suppression of lipolysis in rats and mice [20]. In at least one human study the rate of in vivo lipolysis was not affected by olanzapine or haloperidol treatment in healthy subjects [16]. It is tempting to speculate therefore that olanzapine-induced insulin resistance cause an acute increase in fatty acid oxidation since excessive fat oxidation is linked to tissue insulin resistance [46], [47] while at the same time suppressing or preventing an increase lipolysis normally associated with insulin resistant states [20]. Future mechanistic studies in animal models may be particularly useful in determining the cause of this reproducible effect initially described in clinical studies.

### Effects of Olanzapine on Triglycerides and Cholesterol

Chronic antipsychotic therapy is associated with elevated triglycerides and hypercholesterolemia, though most studies have focused on measurements of insulin resistance and glucose intolerance. In rodents, acute olanzapine-induced insulin resistance is associated with decreased triglycerides, consistent with drug-stimulation of fatty fuel oxidation. Vidarsdottir and colleagues showed that eight days of olanzapine, but not haloperidol, blunted the insulin-mediated decreases in triglycerides during a hyperinsulinemic-euglycemic clamp [16]. Fasting triglycerides, though, were unchanged in their study in contrast to the elevated triglycerides that were observed in the current study.

Studying the cholesterol triglyceride effects of antipsychotics is hindered in rodents, given that rodents do not have the same susceptibilities to dyslipidemia and atherosclerosis as humans [48]. Regardless, it is clear that chronic antipsychotic treatment is associated with increased total and LDL cholesterol as well as decreases in HDL cholesterol clinically, reviewed in: [19], [40]. The current study examined three cholesterol species with standard lipid profiling that is routinely used in clinical laboratories. We did not expect to observe alterations in cholesterol profiles in the olanzapine group; however, the small but significant decline in HDL raises the possibility that antipsychotics may have acute adverse effects on cholesterol/lipid profiles as well. Better-designed, more thorough clinical studies looking specifically at plasma cholesterol species are needed to confirm rapid effects of antipsychotics on cholesterol.

### Generalizability and Limitations of the Trial

The purpose of the current study was to detect and validate observations made from animal models in healthy human volunteers and thus provide ‘proof of concept’ that animal models provide reliable information about the nature of the metabolic effects of atypical antipsychotics. The strength of the trial is in its design – prospective, double blind, randomized, and placebo-controlled with crossover – to maximize sensitivity for detecting acute metabolic changes. As within- and between-subject variability is inherent in clinical studies, we also opted to recruit a very narrowly defined group of volunteers to maximize the sensitivity of the trial. This design begs the question of how generalizable the current findings are to other populations, especially those patients receiving olanzapine therapeutically. This is obviously a limitation of the current study, as it was not well designed for this purpose. The sex-dependence of the olanzapine-induced hyperphagia and weight gain in animals was also not examined in the current study, which may or may not exist in humans [49]. With evidence of the acute metabolic effects of olanzapine, these and other more specific questions can be addressed in future studies.

The safety of the volunteers participating in the study was particularly scrutinized by the investigators and the IRB prior to study approval, since the inherent risks of side effects of receiving olanzapine without any direct therapeutic benefit is not ethical. The rapid time course of these effects in animals is what enabled us to justify the short-term (3 day) exposure of otherwise healthy volunteers with olanzapine. The most serious effects of olanzapine (e.g. neuroleptic malignant syndrome, acute dystonic reaction) were not observed in the current study, and were not expected given that these are typically observed after chronic treatment (>6 months) with higher doses of olanzapine in much older populations. Individuals with a history of seizure and cardiac arrhythmias were also excluded to decrease the chances of these adverse events as well, even though risk of arrhythmia or seizure was considered very low at this particular dosage and length of exposure of olanzapine. None of the volunteers in the study experienced any serious side effects during the olanzapine or placebo arms of the trial. However, more than half of the volunteers did subjectively report fatigue and tiredness, along with dry mouth during the olanzapine arm of the study. With the exception of one case of orthostatic hypotension that was particularly debilitating for one volunteer, there were no significant adverse effects of the short-term olanzapine treatment in these otherwise healthy volunteers.

### Conclusion

Overall this study demonstrates that olanzapine has rapid metabolic effects in healthy human volunteers that are detectable by at least the third day of drug administration. Contrary to animal models, serum leptin appears to be increased, rather than decreased and is likely not directly involved with increased caloric intake in patients taking olanzapine. On the other hand, drug effects on glucose tolerance and FFA lowering are similar to those observed in animal models, and thus these models may be useful in elucidating the physiologic basis of these effects in humans. The acute onset of these metabolic effects may permit screening for patient susceptibility to all or some of the adverse metabolic effects of olanzapine prior to initiation of chronic therapy, though future studies in patients are needed to correlate these acute metabolic changes with chronic outcomes.

## Supporting Information

Checklist S1 This is the CONSORT 2010 checklist of information recommended when reporting the results of a clinical trial.
(PDF)

Protocol S1 This is the final Penn State College of Medicine clinical research proposal that was approved by the Institutional Review Board on 6/12/2008 for the studies being published here.
(PDF)
