# Supplementary material for: A Double Blind, Placebo-Controlled, Randomized Crossover Study of the Acute Metabolic Effects of Olanzapine in Healthy Volunteers
Source: PLoS One. 2011 Aug 9;6(8):e22662. doi: 10.1371/journal.pone.0022662 (PMC3153475; doi:10.1371/journal.pone.0022662)
Supplement: Protocol S1 — This is the final Penn State College of Medicine clinical research proposal that was approved by the Institutional Review Board on 6/12/2008 for the studies being published here. (PDF) [file pone.0022662.s002.pdf]

Clinical Research Proposal
The Pennsylvania State University College of Medicine
Milton S. Hershey Medical Center

Title of Project:	A double-blind, placebo-controlled, crossover study examining the acute effects of olanzapine on plasma leptin, glucose tolerance and free fatty acids in healthy volunteers.
Principal Investigator:	Vance L. Albaugh, B.S.
Co-Investigators:	Ravi Singareddy, M.D.<br>Christopher J. Lynch, Ph.D.

I. Specific Aim

To conduct a clinical research study examining the acute effects of olanzapine on plasma leptin, oral glucose tolerance and appetite in healthy volunteers. We will test the *hypotheses* that olanzapine (1) rapidly attenuates plasma leptin and (2) rapidly alters glucose tolerance in humans by conducting oral glucose tolerance tests in healthy subjects after acute olanzapine exposure. To control for non-responders and genetic variability, respectively, we will use a standardized hunger questionnaire and perform double-blinded tests with and without drug treatment (crossover design).

II. Background and Significance

Schizophrenia & Antipsychotics. Antipsychotic medications comprise several groups of drugs used to treat a variety of psychiatric disorders; including schizophrenia, bipolar disorder, acute mania, major depression, delirium as well as other psychoses. Collectively these disorders will affect 3-5% of the population at some point during their lives[1], potentially exposing a significant portion of the population to antipsychotic treatment. Schizophrenia, in particular, is a complex disease marked by combinations of positive symptoms (e.g. visual hallucinations, thought disorders) and negative symptoms (e.g. blunted affect, anhedonia). The pathophysiology of schizophrenia is unknown, however the positive symptoms are thought to result from excessive dopaminergic stimulation in the brain. Until the 1950s, treatment options for schizophrenia were limited and the advent of antipsychotics (e.g. chlorpromazine, haloperidol) represented a significant advancement in psychopharmacology. These first generation compounds possessed good efficacy against the positive symptoms of schizophrenia, predominately because they are dopaminergic antagonists. Unfortunately, these drugs are non-selective and exhibited severe extrapyramidal symptoms (EPS), tardive dyskinesia (TK) and hypotension which decreased quality of life and patient adherence. Aside from these adverse effects, first generation antipsychotics also caused moderate weight gain, though this problem was far outweighed by the debilitating EPS and TK[2]. Second generation antipsychotics came onto the U.S. market in 1988 with the introduction of Clozapine (Clozaril®), a powerful antipsychotic that alleviated both positive and negative symptoms of schizophrenia. Currently, clozapine therapy is limited to treatment-resistant patients due to the incidence of life-threatening agranulocytosis (~1%). However, the development of other second generation antipsychotics

soon followed, including Eli Lilly's olanzapine (Zyprexa®) in the mid-1990s. These newer drugs revolutionized the treatment of psychotic disorders because they lacked EPS and possessed equal or better efficacy against the positive and negative symptoms of schizophrenia. Pharmacologically, these 'atypical' compounds have lower affinities for D2-like dopamine receptors and higher affinities for serotonin-2A (5-HT_{2A}) receptors. As the popularity and off-label uses of olanzapine and other new 'atypical' drugs increased, so did evidence suggesting possible metabolic side effects; including ketoacidosis[3-7], dyslipidemia[8, 9], severe weight gain[10-13], hyperglycemia[14-16] and even death[17-19]. Meta-analysis has confirmed that olanzapine and clozapine cause the most significant weight gain and other metabolic effects[20]. Indeed, the Food & Drug Administration (2003) currently mandates that labels of atypical antipsychotic drugs include warnings to patients about the dangerous side effects of diabetic ketoacidosis and hyperosmolar coma. Additionally, the American Diabetes Association, American Psychiatric Association, American Association of Clinical Endocrinologists, and North American Association for the Study of Obesity issued a consensus statement recommending guidelines for monitoring weight gain, glucose tolerance and hypertension in these patients[21]. Despite these obvious risks, atypical antipsychotics continue to be widely prescribed because of their unmatched efficacy, which outweighs their metabolic risks[22]. The mechanism(s) of these metabolic side effects remains elusive; however, identifying these mechanism(s) could provide a useful strategy to reduce adverse events and to develop drugs with fewer side effects.

Clinical Investigation of Antipsychotic-Induced Obesity. The metabolic side effects of atypical antipsychotics are numerous, with obesity and diabetes merely being the most well-known[23-27]. Several studies have aimed at elucidating weight gain mechanisms, but prospective studies and clinical research in volunteers has be less than fruitful[28-31]. Prospective studies in patient populations comparing weight gain among individuals have clearly demonstrated the propensities for different atypical antipsychotics to cause weight gain in patient populations, but none of these studies has been able to determine any probable weight gain mechanisms[32-35]. One of the problems with these previous studies – both clinical and patient trials – is that results are complicated because of comorbid conditions, non-drug naïve patient populations. Nearly all clinical studies to date have examined the effects of these antipsychotics after short- or long-term use; this is similar to the study of animal models of this side effect until recently. One of the problems with this research design is the nature of the long term effects. Several studies have examined changes in adipokines, such as leptin, because of the increases of adipose tissue mass in individuals given these medications[36]. Because of the difficulty of determining the mechanism of these side effects, some trials have alternatively focused on interventions designed to attenuate weight gain[37]. Overall, the clinical study of these side effects has given limited insight to this side effect and new directions are needed.

Emerging Data from Preclinical Studies. Several rodent models have been developed[38-41], though none of these models has identified the cause of hyperphagia or weight gain[42]. Initially, rats did not appear to be a suitable model for the weight gain side effect[43, 44]; with studies reporting that drug effects in males were limited to transient increases (~30 min) in food intake without changes in daily consumption[45]. In striking contrast, however, female rats were shown to exhibit a rapid and severe weight gain with atypical antipsychotic treatment (e.g. olanzapine, risperidone and sulpiride). This weight gain is secondary to hyperphagia and increased fat pad mass without changes in basal metabolic rate[46]. In our studies with female rats, olanzapine

administration caused significantly higher body weight within ~2-3 days of treatment[47]. Because antipsychotic-induced weight gain is caused by excessive food intake, our goal is to identify the mechanism of hyperphagia. Data from chronic studies revealed that hyperphagia never began until ~24 hours after the first dose of drug with stronger effects on subsequent days. Because of this delay, we hypothesized that acute metabolic changes may be detectable before the onset of hyperphagia that might explain it. Subsequently, animals were challenged with an oral glucose tolerance test after ~24 hours of treatment to see whether or not glucose tolerance and/or glycemia were affected. Our results were unexpected, with improvements in glucose tolerance after acute olanzapine treatment. Moreover, olanzapine-treated rats had decreased basal plasma concentrations of glucose, insulin and leptin – known satiety factors with strong influences on food intake. Leptin, arguably the most important of the three, is secreted by adipocytes and serves as a signal of peripheral energy stores to the hypothalamus, which controls food intake[48]. Decreases or losses (e.g. ob/ob mice, Zucker fatty rats) in the leptin signal to the hypothalamus is interpreted as starvation by the brain, leading to hyperphagia and, in the case of a complete loss, to obesity. These preliminary findings suggest that understanding the mechanism of the acute drug effects will lead to a better understanding of their chronic metabolic effects. Additionally, it is imperative that the correct model for the human side effect be determined, because male and female rats have opposing metabolic responses to acute administration of olanzapine. Female rats rapidly gain weight and better mimic the weight gain side effect, though male rats rapidly develop insulin resistance which is another rapid complication in some patients. More importantly, the insulin resistance in male rats develops within 24hrs of treatment, independent of changes in food intake or body composition. Conducting a clinical study to determine which rat sex correctly models this effect is warranted and needed to further any fruitful basic investigation of this side effect. The crossover design of the proposed research offers sufficient power to detect a sexual dimorphism, if one does exist in humans, and determine the acute effects of olanzapine clinically.

Summary: Our laboratory has been interested in the metabolic effects of atypical antipsychotics for the past several years. Most of the human and animal studies predating our work focused on the long-term effects of these drugs in an effort to determine how the drugs cause weight gain and diabetes. Our group has identified rapid metabolic effects in rodents and we think these effects may underlie the development of obesity and diabetes clinically. The purpose of this study is to determine if healthy volunteers exhibit similar metabolic side effects to those observed in rodents.

III. Research Design, Methods and Human Subjects

1. **Population to be Studied:** The study will use 16 healthy volunteers without any DSM-IV TR Axis-I psychiatric disorders or other chronic diseases/conditions. Every effort will be made to recruit equal gender and racial/ethnic diversity. Based on recent census data, though, the surrounding area of Dauphin County is only 17% African American and 2% Asian. In terms of minorities, we are only expecting to enroll individuals of African American and Asian descent, but other minority groups will be encouraged to participate in the study.

2. **Inclusion/Exclusion Criteria:** Eligible individuals are between the ages 18-30 with body mass indexes (BMIs) between 18.5-25 kg/m^2. The reason for the narrow range of

BMI is to recruit healthy volunteers and exclude any confounding factors associated with BMIs greater than 25. BMIs less than 18.5 are also considered unhealthy, and thus we are excluding these individuals as well. The higher the BMI of the individual, the higher the adipose tissue mass (most likely). Because leptin circulates in the plasma after it is released from adipocytes, the plasma concentration correlates with the total adipose mass. These recruitment criteria are necessary to exclude individuals with high BMIs (high adipose mass) that may have leptin or insulin resistance, potentially confounding data analysis.

Inclusion Criteria	Exclusion Criteria
• Healthy Volunteer<br>• Aged 18-30 years<br>• Body Mass Index (BMI) between 18.5-25kg/m^2<br>• Able to give informed consent	• Any DSM-IV TR Axis I psychiatric disorder (except nicotine dependence)<br>• Presence of any medical disorder that may confound the assessment of relevant biologic measures, including: significant organ system dysfunction, metabolic diseases, type 1 diabetes mellitus, type 2 diabetes mellitus, pregnancy, endocrine disease, coagulopathy, clinically significant anemia, or acute infection;<br>• Subjects who have taken any antipsychotic medication within the last 6 months;<br>• Subjects taking certain prescription medications (as determined by Dr. Singareddy on a case by case basis).<br>• Personal or family history of seizures and/or cardiac arrhythmias.

3. **Study Design:** The study design is a double-blind, placebo-controlled, crossover experiment.

4. **Recruitment and consent process:** Subjects will be recruited through posted fliers in the College of Medicine, Milton S. Hershey Medical Center and other satellite clinics in the surrounding area to attract a racially/ethnically diverse population. Interested individuals will be instructed to call the GCRC for more information and to answer a few questions over the phone with a trained GCRC staff person to be screen for eligibility. For individuals that meet the inclusion and exclusion criteria, a consent form that clearly explains the goals and procedures of the study will be sent to the individual or otherwise picked up in the GCRC. Risks as well as procedures to protect the subjects from adverse effects will be explained in lay words. Informed consent will be obtained by the principal investigator after the subject has had the study described orally and the subject has had a chance to read the consent form and have all questions answered. Subjects will be given a copy of the consent form to take home.

5. **Procedures to be followed:**

Randomization & General Procedures: Olanzapine and placebo tablets will be packaged and randomized prior to the start of the study by the Investigational Drug Service at the Milton S. Hershey Medical Center. A copy of the 'key' that will be used in cases of emergency unblinding will be kept by Dr. Singareddy in his office. Volunteers will be given olanzapine (10 mg) or placebo tablets to be taken once a day for 3 days before each study visit. On arrival at the GCRC on the first testing date, a general medical questionnaire will be completed. Volunteers capable of becoming pregnant will be given a urine dipstick pregnancy test when they come to the GCRC to pick up their first package of study drug.

Appetite Survey: On both testing days the research volunteer will be given an appetite survey before beginning the oral glucose tolerance test. The appetite survey is conducted using a visual analog scale (VAS). This is a simple test to gauge the participant's level of hunger and is used in many laboratories that study human weight loss and dietary manipulation. The participant will read a question on a single piece of paper. For example, "How hungry do you feel right now?" Below the question is a 100mm line with two extreme answers on the far left and far right ends of the solid line. Examples of extreme answers for this particular question would be, "Not hungry at all" on the far left end and "Extremely hungry" printed on the far right. After the question is read the participant will indicate with a single vertical line how he/she feels.

Oral Glucose Tolerance Test (OGTT): Before each of the testing dates volunteers will be asked to eat a high carbohydrate diet and will be instructed on how to eat a high carbohydrate diet when they come to the GCRC to pick up their study medication. After an overnight fast of 10-14 hours, subjects will receive 75 grams of an oral glucose solution at time zero (t = 0) and blood will be sampled every 30 minutes for two hours (t = 30, 60, 90, 120) for determination of glucose and insulin levels. An IV is inserted, if the patient desires, to avoid multiple phlebotomy and normal saline is run at KVO during the study.

Leptin and Free Fatty Acid Assays: Additional blood samples will be drawn with the IV access obtained for the OGTT. Leptin will be measured with a radioimmunoassay for human leptin. A free fatty acid assay will also be conducted by the Core Endocrine Lab.

6. **Primary and Secondary Endpoints:** The primary endpoint for the study and for which power analysis has been calculated is change in plasma leptin concentration. Secondary endpoints include the changes in the area under the glucose and/or insulin curves, changes in free fatty acids and appetite.

7. **Statistics and Sample Size Justification:** The primary measurement for the study is the change in plasma leptin concentration – placebo vs. olanzapine. Using a conservative estimate of within-subject correlation, assuming a modest correlation benefit for the cross-over design and using Standard Deviations estimated from the figure (n=10) from the previous rodent study[47], a total of 16 subjects will have 90% power to detect a 40% effect size. The secondary endpoints that will be analyzed are Area under the Glucose and Insulin curves (AUC) from the oral glucose tolerance test and the plasma free fatty

acid concentrations. The mean $AUC_{glucose}$, $AUC_{insulin}$, plasma leptin and free fatty acid concentrations will be compared using Student's t-test ($\alpha = 0.05$).

8. **Risks and Discomforts:** The risks of this study are minimal, especially because the subjects will only be receiving three doses (Total dose = 30 mg) of olanzapine over a three day period. **(1) OLANZAPINE:** Risks of this amount of olanzapine may include the following: constipation, dry mouth, feeling weak, upset stomach, dystonia, akathisia, tremors (shakes), dizziness, insomnia, blurry vision, irregular heartbeat, drowsiness and postural hypotension. There is also the potential for seizures; however the chance is small and the co-sponsor has not observed any olanzapine-related seizures in his >3 years of post-training clinical experience. According to the co-sponsor, no prophylactic measures are taken clinically for this rare effect. However, to take maximal precautions and further decrease the possibility of seizures, volunteers will a family or personal history of seizures will be will excluded. Other risks include neuroleptic malignant syndrome and tardive dyskinesia. Volunteers should not drink alcohol when taking olanzapine. **(2) BLOOD DRAW:** Risks of blood draw: there is a risk of development of hematoma and/or phlebitis. This will be minimized by having the blood draws performed by an experienced phlebotomist.

   Protection Against Risks: GCRC support staff regularly conduct oral glucose tolerance tests and will be responsible for proper administration of these tests and monitoring for any adverse effects that might result while in the GCRC. Sterile techniques will be used to minimize any risk of infection during blood draws, which will be done by an experienced phlebotomist. Subjects will be free to withdraw from the study at any time. All data remains confidential. Medical care is immediately available if needed.

9. **Benefits:** (*To Study Participants*) There are no direct benefits to study participants other than gaining the knowledge of the glucose tolerance test results, which could reveal diabetes or pre-diabetes. If a volunteer does have impaired glucose tolerance it may alert those individuals in his/her family that they too should discuss screening with their family physician, thus benefiting the volunteer and his/her family.

   (*Benefits to Society*) If similar findings are demonstrated in humans compared with rodent studies, it would suggest that weight gain and the metabolic effects of the drugs are compensatory responses to the acute drug effects. This is important in determining the mechanism of these effects and especially important for future research in humans which may be more fruitful if drug effects are studied acutely. This study may reveal acute metabolic effects in humans similar to rats which have been implicated in the mechanism of drug-induced obesity. Given the low risk, the benefits of the knowledge gained though the completion of this research greatly outweigh the risks of the study.

10. **Safety and Confidentiality:** All data in the experiment are for research purpose only. All data are labeled with a unique identifier and de-identified after all assays are successfully completed. Data obtained during the study are confidential and kept in locked cabinets in C4757. Data stored electronically are password-protected. All personal identifiers will be removed from data presented in scientific meetings or peer-

reviewed publications. Only investigators listed on the protocol will have access to the data obtained before it is de-identified.

11. **Data Safety and Monitoring Plan**: A Data Safety and Monitoring Plan has been submitted to the General Clinical Research Center and is attached. All data collected during the procedures outlined in the protocol will be kept confidential and either on password protected computer in the PI's laboratory (C4757) or in a cabinet in the PI's locked laboratory. As the data is analyzed personal identifiers (i.e. initials) will be removed to keep confidentiality. All data will be stored according to federal guidelines and then destroyed.

    Other data collected as a part of the prescreening process will be kept in the PI's locked laboratory and will only be available to the PI's research team, or the General Clinical Research Center, if the subject agrees to have their information shared.

12. **Compensation:** Volunteers will be compensated $150 for their participation in the study. ($50 for completion of the first week and $100 for completion of the second week) This reimbursement scheme is to ensure that individuals will return to the GCRC for their second test date. Because of the interindividual variability in circulating plasma leptin concentration, it is scientifically necessary to obtain glucose tolerance test results in every individual in the presence and absence of olanzapine.

13. **Drugs, Devices or Biologics:** Olanzapine is an FDA-approved medication and is the only drug that will be administered in this study. Olanzapine was initially approved for the treatment of schizophrenia on September 30, 1996. It is currently approved for the treatment of bipolar disorder and is used off-label for numerous psychiatric illnesses including, but not limited to; anxiety spectrum disorders, anorexia nervosa, Tourett's syndrome and stuttering.

IV. Investigator Qualifications and Roles in the Conduct of the Study

The principal investigator has four years of basic science research. The co-investigators listed in the protocol are mentoring the principal investigator as this is his first clinical research endeavor. Of these individuals, Dr. Singareddy, a board-certified psychiatrist and Assistant Professor, will be overseeing the study and has greater than 6 years of clinical research experience. Dr. Singareddy will be specifically involved in safety monitoring and consenting of individuals, data interpretation and subject recruitment. The other co-investigator, Dr. Lynch, has greater than four years of research experience involving human subjects and over 25 years of basic science research experience. Dr. Lynch will be involved in data interpretation.

V. Facilities

This study will be conducted in the General Clinical Research Center at the Milton S. Hershey Medical Center.

VI. References

1. **Jibson MD**. *Overview of Antipsychotic Medications*. 2006 August 3, 2006 [cited; Available from: http://www.uptodateonline.com/utd/content/topic.do?topicKey=psychiat/13184.
2. **Allison DB and Casey DE**. Antipsychotic-induced weight gain: a review of the literature. J Clin Psychiatry, 2001. 62 Suppl 7: p. 22-31.
3. **Rigalleau V*, et al.*** Diabetes as a result of atypical anti-psychotic drugs--a report of three cases. Diabet Med, 2000. 17(6): p. 484-6.
4. **Gatta B*, et al.*** Diabetic ketoacidosis with olanzapine treatment. Diabetes Care, 1999. 22(6): p. 1002-3.
5. **Kyriazis IA*, et al.*** Diabetic ketoacidosis and severe dyslipidemia in an adult psychotic man with olanzapine treatment. J Clin Psychopharmacol, 2006. 26(1): p. 92-4.
6. **Straker D*, et al.*** Near fatal ketoacidosis with olanzapine treatment. Psychosomatics, 2002. 43(4): p. 339-40.
7. **Ragucci KR and Wells BJ**. Olanzapine-induced diabetic ketoacidosis. Ann Pharmacother, 2001. 35(12): p. 1556-8.
8. **de Leon J*, et al.*** A clinical study of the association of antipsychotics with hyperlipidemia. Schizophr Res, 2007. 92(1-3): p. 95-102.
9. **Olfson M*, et al.*** Hyperlipidemia following treatment with antipsychotic medications. Am J Psychiatry, 2006. 163(10): p. 1821-5.
10. **Bryden KE and Kopala LC**. Body mass index increase of 58% associated with olanzapine. Am J Psychiatry, 1999. 156(11): p. 1835-6.
11. **eder-Ischia U*, et al.*** Olanzapine-induced weight gain and disturbances of lipid and glucose metabolism. Essent Psychopharmacol, 2005. 6(2): p. 112-7.
12. **Haapasalo-Pesu KM and Saarijarvi S**. Olanzapine induces remarkable weight gain in adolescent patients. Eur Child Adolesc Psychiatry, 2001. 10(3): p. 205-8.
13. **Karagianis J**. Olanzapine and weight gain. Can J Psychiatry, 2000. 45(5): p. 493.
14. **Laimer M*, et al.*** Olanzapine-induced hyperglycemia: role of humoral insulin resistance-inducing factors. J Clin Psychopharmacol, 2005. 25(2): p. 183-5.
15. **Fertig MK*, et al.*** Hyperglycemia associated with olanzapine. J Clin Psychiatry, 1998. 59(12): p. 687-9.
16. **Koller EA and Doraiswamy PM**. Olanzapine-associated diabetes mellitus. Pharmacotherapy, 2002. 22(7): p. 841-52.
17. **Avella J*, et al.*** Fatal olanzapine-induced hyperglycemic ketoacidosis. Am J Forensic Med Pathol, 2004. 25(2): p. 172-5.
18. **Meatherall R and Younes J**. Fatality from olanzapine induced hyperglycemia. J Forensic Sci, 2002. 47(4): p. 893-6.
19. **Torrey EF and Swalwell CI**. Fatal olanzapine-induced ketoacidosis. Am J Psychiatry, 2003. 160(12): p. 2241.
20. **Allison DB*, et al.*** Antipsychotic-induced weight gain: a comprehensive research synthesis. Am J Psychiatry, 1999. 156(11): p. 1686-96.
21. **American Diabetes Association APA, American Society of Clinical Endocrinologists, North American Association for the Study of Obesity.** Consensus development conference on antipsychotic drugs and obesity and diabetes. Diabetes Care, 2004. 27(2): p. 596-601.
22. **Newcomer JW**. Second-generation (atypical) antipsychotics and metabolic effects: a comprehensive literature review. CNS Drugs, 2005. 19 Suppl 1: p. 1-93.
23. **Newcomer JW**. Metabolic considerations in the use of antipsychotic medications: a review of recent evidence. J Clin Psychiatry, 2007. 68 Suppl 1: p. 20-7.
24. **Newcomer JW**. Antipsychotic medications: metabolic and cardiovascular risk. J Clin Psychiatry, 2007. 68 Suppl 4: p. 8-13.
25. **Newcomer JW**. Metabolic risk during antipsychotic treatment. Clin Ther, 2004. 26(12): p. 1936-46.
26. **Newcomer JW**. Abnormalities of glucose metabolism associated with atypical antipsychotic drugs. J Clin Psychiatry, 2004. 65 Suppl 18: p. 36-46.
27. **Lindenmayer JP*, et al.*** Changes in glucose and cholesterol levels in patients with schizophrenia treated with typical or atypical antipsychotics. Am J Psychiatry, 2003. 160(2): p. 290-6.
28. **Sentissi O*, et al.*** Leptin and Ghrelin Levels in Patients With Schizophrenia During Different Antipsychotics Treatment: A Review. Schizophr Bull, 2007.
29. **Himmerich H*, et al.*** Ghrelin plasma levels during psychopharmacological treatment. Neuropsychobiology, 2005. 52(1): p. 11-6.

30. **Melkersson K and Dahl ML**. Adverse metabolic effects associated with atypical antipsychotics: literature review and clinical implications. Drugs, 2004. 64(7): p. 701-23.
31. **Haupt DW and Newcomer JW**. Hyperglycemia and antipsychotic medications. J Clin Psychiatry, 2001. 62 Suppl 27: p. 15-26; discussion 40-1.
32. **Saddichha S, *et al.*** Effect of olanzapine, risperidone, and haloperidol treatment on weight and body mass index in first-episode schizophrenia patients in India: a randomized, double-blind, controlled, prospective study. J Clin Psychiatry, 2007. 68(11): p. 1793-8.
33. **Perez-Iglesias R, *et al.*** A 12-week randomized clinical trial to evaluate metabolic changes in drug-naive, first-episode psychosis patients treated with haloperidol, olanzapine, or risperidone. J Clin Psychiatry, 2007. 68(11): p. 1733-40.
34. **Hosojima H, *et al.*** Early effects of olanzapine on serum levels of ghrelin, adiponectin and leptin in patients with schizophrenia. J Psychopharmacol, 2006. 20(1): p. 75-9.
35. **Bushe C and Leonard B**. Association between atypical antipsychotic agents and type 2 diabetes: review of prospective clinical data. Br J Psychiatry Suppl, 2004. 47: p. S87-93.
36. **Haupt DW, *et al.*** Plasma leptin and adiposity during antipsychotic treatment of schizophrenia. Neuropsychopharmacology, 2005. 30(1): p. 184-91.
37. **Wu RR, *et al.*** Lifestyle intervention and metformin for treatment of antipsychotic-induced weight gain: a randomized controlled trial. Jama, 2008. 299(2): p. 185-93.
38. **Arjona AA, *et al.*** An animal model of antipsychotic-induced weight gain. Behav Brain Res, 2004. 152(1): p. 121-7.
39. **Pouzet B, *et al.*** Chronic treatment with antipsychotics in rats as a model for antipsychotic-induced weight gain in human. Pharmacol Biochem Behav, 2003. 75(1): p. 133-40.
40. **Baptista T, *et al.*** Comparative effects of the antipsychotics sulpiride or risperidone in rats. I: bodyweight, food intake, body composition, hormones and glucose tolerance. Brain Res, 2002. 957(1): p. 144-51.
41. **Cope MB, *et al.*** Antipsychotic drug-induced weight gain: development of an animal model. Int J Obes (Lond), 2005. 29(6): p. 607-14.
42. **Goudie AJ, *et al.*** Antipsychotic-induced weight gain. Diabetes Obes Metab, 2005. 7(5): p. 478-87.
43. **Benvenga MJ, Leander, J.D.** Increased food consumption by clozapine, but not by olanzapine, in satiated rats. Drug Development Research, 1997. 41(1): p. 48-50.
44. **Baptista T, *et al.*** Effects of long-term administration of clozapine on body weight and food intake in rats. Pharmacol Biochem Behav, 1993. 45(1): p. 51-4.
45. **Lee MD and Clifton PG**. Meal patterns of free feeding rats treated with clozapine, olanzapine, or haloperidol. Pharmacol Biochem Behav, 2002. 71(1-2): p. 147-54.
46. **Coccurello R, *et al.*** Chronic administration of olanzapine induces metabolic and food intake alterations: a mouse model of the atypical antipsychotic-associated adverse effects. Psychopharmacology (Berl), 2006. 186(4): p. 561-71.
47. **Albaugh VL, *et al.*** Hormonal and metabolic effects of olanzapine and clozapine related to body weight in rodents. Obesity, 2006. 14(1): p. 36-51.
48. **Schwartz MW, *et al.*** Central nervous system control of food intake. Nature, 2000. 404(6778): p. 661-71.
